# Porcine Erythrocyte–PRRSV Interactions: Implications for Targeted Nanodrug Delivery

**DOI:** 10.3390/vetsci13060555

**Published:** 2026-06-04

**Authors:** Wei Yin, Jingze Li, Haoxiang Yao, Jingyi Qiao, Jia Zhong, Yaogui Sun, Hongquan Li, Kuohai Fan, Zhenbiao Zhang, Na Sun, Panpan Sun, Huizhen Yang, Jianzhong Wang

**Affiliations:** Shanxi Key Laboratory for Modernization of TCVM, College of Veterinary Medicine, Shanxi Agricultural University, Jinzhong 030801, China; 202531036@stu.sxau.edu.cn (J.L.); 202531098@stu.sxau.edu.cn (H.Y.); yinwei126yinyue@126.com (J.Q.); zhongjia3294@163.com (J.Z.); dkyypb@163.com (Y.S.); livets@163.com (H.L.); fkhyxj@163.com (K.F.); zbzhangvet@sxau.edu.cn (Z.Z.); snzh060511@126.com (N.S.); 18404966309@163.com (P.S.); hzyang2020@163.com (H.Y.); jianzhongwang@cau.edu.cn (J.W.)

**Keywords:** porcine CR1-like, PAMs, PRRSV, mannose, matrine

## Abstract

Objective: To establish a porcine complement receptor type 1-like (CR1-like)-mediated targeted anti-porcine reproductive and respiratory syndrome virus (PRRSV) nanodrug delivery system by investigating interactions between erythrocytes from Landrace piglets (both male and female), PRRSV, and anti-PRRSV nanodrugs. Methods: Optimal conditions for PRRSV sensitization with fresh porcine serum were determined. CR1-like-dependent immune adhesion of porcine erythrocytes to sensitized PRRSV was verified by immunofluorescence, electron microscopy, qPCR, and Western blot. The effect of this adhesion on PRRSV infection of porcine alveolar macrophages (PAMs) was studied using a flow chamber system. Mannose-modified matrine nanoliposomes (MMLNPs) were prepared, characterized, and evaluated for cytotoxicity, targeting ability, and in vitro antiviral activity. Results: PRRSV was optimally sensitized by incubation with fresh porcine serum at 37 °C for 2 h. Porcine erythrocytes specifically adhered to sensitized PRRSV via CR1-like, significantly promoting PRRSV infection of PAMs. Stable, uniform-sized MMLNPs showed no cytotoxicity, targeted PAMs via CR1-like, and exhibited superior antiviral activity to free matrine. Conclusions: CR1-like-mediated immune adhesion is a critical mechanism for PRRSV infection of PAMs. Harnessing this natural pathway enables efficient targeted delivery of matrine nanoliposomes to PAMs, providing a promising translational strategy for PRRSV control.

## Simple Summary

Porcine reproductive and respiratory syndrome (PRRS), induced by the PRRS virus (PRRSV), predominantly targets porcine alveolar macrophages (PAMs) and results in substantial economic losses within the global swine industry. The efficacy of current antiviral drugs is hindered by inadequate targeting and limited intracellular effectiveness. This investigation refined the in vitro sensitization conditions for PRRSV using fresh serum from Landrace piglets, determining that incubation at 37 °C for 2 h was optimal. Our findings demonstrated that porcine erythrocytes specifically adhere to serum-sensitized PRRSV via the porcine complement receptor type 1-like (CR1-like), significantly enhancing PRRSV infection of PAMs. Additionally, we developed mannose-modified matrine nanoliposomes (MMLNPs) characterized by uniform size, stability, and lack of cytotoxicity. MMLNPs were effectively targeted to PAMs by porcine erythrocytes through CR1-like-mediated immune adhesion, displaying superior and more sustained anti-PRRSV activity compared to free matrine. This study introduces an innovative strategy for the prevention and treatment of PRRS.

## 1. Introduction

Porcine reproductive and respiratory syndrome (PRRS), caused by PRRS virus (PRRSV), is one of the most devastating infectious diseases in the global swine industry, leading to severe reproductive disorders in sows and respiratory failure in piglets, with annual economic losses reaching billions of dollars [1,2]. PRRSV primarily targets porcine alveolar macrophages (PAMs) and establishes persistent infection through immune evasion mechanisms [3,4]. Current vaccines and antiviral drugs have limited efficacy due to rapid viral mutation and poor intracellular delivery to PAMs [5]. Matrine, a major quinolizidine alkaloid isolated from Sophora flavescens, has been demonstrated to inhibit PRRSV replication by suppressing N protein expression, Nsp9 activation, and NLRP3 inflammasome-mediated inflammation [6,7,8,9,10]. However, free matrine suffers from low bioavailability and insufficient accumulation in PAMs, which severely restricts its clinical application. Erythrocyte-based drug delivery systems leverage natural immune adhesion mechanisms to achieve targeted delivery to phagocytes, offering superior biocompatibility and circulation longevity compared to synthetic carriers. This inspired us to explore whether the porcine erythrocyte–PRRSV interaction could be repurposed for targeted anti-PRRSV therapy.

Erythrocytes exert critical innate immune functions through complement receptor type 1 (CR1), which specifically binds to C3b/C4b-opsonized pathogens or immune complexes (ICs) and transports them to the liver and spleen for clearance by phagocytes. Multiple viruses exploit complement-dependent pathways to interact with erythrocytes, including HCV, SARS-CoV, HIV, PEDV and Zika virus [11,12,13,14,15,16]. However, whether PRRSV can adhere to porcine erythrocytes remains unknown [17].

Porcine erythrocytes express a functional homolog of human CR1, designated porcine complement receptor type 1-like (CR1-like) [18,19,20]. The full-length cDNA of porcine CR1-like (4391 bp) has been cloned and sequenced (GenBank: KF286608), and bioinformatics analysis revealed that it contains 19 complement control protein (CCP) modules, with CCP 1–3 and CCP 8–10 responsible for C4b and C3b binding, respectively [21,22,23]. Our group previously developed a monoclonal antibody against porcine CR1-like [24,25] and demonstrated that CR1-like is expressed on both erythrocytes and PAMs [26]. We further showed that PAMs can remove C3b-opsonized pathogens from erythrocyte surfaces via CR1-like, and that CR1-like mediates antibody-dependent enhancement (ADE) of PRRSV infection through the Rac-1/Cdc42-PAK1-LIMK1-Cofilin signaling pathway [27]. These findings suggest that CR1-like plays a central role in the interaction between erythrocytes, pathogens, and PAMs, but its specific function in PRRSV transmission remains unclear. Our previous work focused on CR1-like-mediated antibody-dependent enhancement of PRRSV infection in static culture systems. In contrast, this study investigates the dynamic erythrocyte–PRRSV interaction under physiological shear stress and pioneers the application of this mechanism for targeted nanodrug delivery, representing a significant extension of our prior research.

Erythrocytes are ideal natural drug carriers due to their high biocompatibility, long circulation half-life (up to 120 days), and inherent expression of CD47, which prevents phagocytosis by the mononuclear phagocyte system (MPS) [28,29,30]. Various therapeutic agents, including enzymes, steroids, and nanoparticles, have been successfully delivered using erythrocyte-based systems [31,32,33]. However, most existing erythrocyte drug delivery systems rely on non-specific surface modification, which may compromise erythrocyte function and targeting efficiency. Given the specific binding between CR1-like and C3b-opsonized particles, we hypothesized that this natural immune adhesion mechanism could be exploited to develop a targeted nanodrug delivery system for PRRSV treatment.

In this study, we first optimized the in vitro sensitization conditions of PRRSV with fresh porcine serum and confirmed that porcine erythrocytes adhere to serum-sensitized PRRSV via CR1-like. We then demonstrated that this CR1-like-mediated immune adhesion significantly enhances PRRSV infection of PAMs under physiological shear stress, revealing a previously unrecognized mechanism of PRRSV systemic dissemination. Building on this fundamental discovery, we hypothesized that we could hijack this natural virus transmission pathway to deliver antiviral drugs specifically to PAMs—the primary target cells of PRRSV. To test this hypothesis, we prepared mannose-modified matrine nanoliposomes (MMLNPs) and evaluated their targeting efficiency and antiviral activity when delivered by porcine erythrocytes. This study establishes a direct link between viral pathogenesis mechanisms and targeted drug delivery strategies, providing a promising platform for the treatment of macrophage-tropic viral infections.

## 2. Animals and Materials

A total of 6 thirty-day-old PRRSV antigen–antibody double-negative Landrace piglets (both male and female, body weight 8–10 kg, Wujiabao Pig Farm, Taigu, Jinzhong, China) were used in this study. All piglets were acclimatized for 24 h in a standard barrier environment (temperature 25 ± 2 °C, relative humidity 50–60%, 12 h light/dark cycle) with free access to food and water; no environmental enrichment was provided. All animal procedures were approved by the Laboratory Animal Ethics Committee of Shanxi Agricultural University (SXAU-EAW-2022P.UP.002015148, Approval Date: 5 February 2022). Piglets were humanely euthanized by intravenous injection of sodium pentobarbital (100 mg/kg body weight) to minimize pain and suffering. Inclusion and exclusion criteria for animals were established a priori: only piglets negative for both PRRSV antigen and antibody were included; no animals were excluded from the study. PRRSV JS-1 strain was isolated from a PRRSV-positive pig farm in Jiangsu Province, China, and preserved in our laboratory. The virus was propagated and titrated in Marc-145 cells as previously described [10].

## 3. Methods

### 3.1. PRRSV Sensitization Condition Optimization

Fresh porcine serum was pooled from the 6 PRRSV-negative Landrace piglets used in this study. Blood was collected into sterile tubes without anticoagulant, allowed to clot at 4 °C for 2 h, and centrifuged at 3000× *g* for 15 min at 4 °C to separate serum. Serum was aliquoted and stored at −80 °C for no more than 2 weeks before use to preserve complement activity. To determine the optimal time for complement-mediated PRRSV sensitization, three groups were set up as shown in Table 1. PRRSV (200 TCID_50_) was incubated with fresh or inactivated porcine serum at 37 °C for 0, 0.5, 1, 2, 3, and 5 h. C3 and CH50 activities in the supernatant were measured by ELISA according to the manufacturer’s instructions. Inactivated porcine serum was prepared by heating fresh serum at 56 °C for 30 min in a water bath. This method was selected as it is the standard procedure for complement inactivation, which specifically destroys the heat-labile complement components while preserving other serum proteins and biological activities.

### 3.2. Detection of Porcine Erythrocyte Immune Adhesion to PRRSV

Porcine erythrocytes were isolated from fresh anticoagulated blood using a peripheral blood erythrocyte isolation kit and adjusted to 2.5 × 10^7^ cells/mL. Five groups were established to verify CR1-like-mediated immune adhesion (Table 2). After incubation with differently treated PRRSV at 37 °C for 30 min, unbound virus was removed by centrifugation and washing. The incubation time of 30 min at 37 °C was selected based on our previous studies demonstrating that this duration is optimal for porcine erythrocyte immune adhesion to complement-opsonized pathogens, and is consistent with standard protocols used in erythrocyte immune adhesion assays [27]. Adhesion was detected by indirect immunofluorescence (FITC-labeled PRRSV), scanning electron microscopy (SEM), transmission electron microscopy (TEM), qPCR (PRRSV N gene), and Western blot (PRRSV N protein). The anti-CR1-like monoclonal antibody used in this study was developed and characterized in our laboratory as previously reported [24,25]. Its specificity has been rigorously verified by Western blot, immunofluorescence assay, and flow cytometry, showing no cross-reactivity with other porcine complement receptors or membrane proteins.

### 3.3. Effect of CR1-like Adhesion on PRRSV Infection of PAMs

A parallel plate flow chamber system (shear force: 5.3 dynes/cm^2^) was used to simulate in vivo blood circulation. This shear force value was selected based on our previously published study using the identical experimental system [21], which demonstrated that 5.3 dynes/cm^2^ represents the physiological shear stress in porcine pulmonary capillaries (1–6 dynes/cm^2^) and ensures stable mobile phase flow without compromising cell viability. PAMs were seeded on poly-L-lysine-coated slides and cultured for 12 h. Four groups were set up, as shown in Table 3. After 1 h of circulation, unbound cells and virus were removed, and intracellular PRRSV N gene copy number was quantified by qPCR.

### 3.4. Preparation and Characterization of MMLNPs

Mannose-modified matrine nanoliposomes (MMLNPs) and blank mannose liposomes (MLNPs) were prepared by thin-film dispersion and liposome extrusion methods. Particle size, polydispersity index (PDI), and zeta potential were measured by dynamic light scattering. Encapsulation efficiency was determined by UV spectrophotometry at 212 nm. To eliminate potential interference from liposomal excipients, blank mannose liposomes (MLNPs) of the same composition were used as the baseline control. A differential spectrophotometry method was employed, where the absorbance of MMLNPs was measured against an equivalent concentration of MLNPs to subtract background absorption. All measurements were performed in triplicate to ensure accuracy. Morphology was observed by TEM. Cytotoxicity to PAMs was evaluated by CCK-8 assay at concentrations ranging from 0.0625 to 2 mg/mL.

Erythrocyte adhesion to MMLNPs was detected by SEM after incubation with fresh or inactivated serum-sensitized MMLNPs (1 mg/mL) at 37 °C for 30 min. PAMs targeting was verified by TEM after 1 h of co-incubation in the flow chamber system.

### 3.5. In Vitro Antiviral Activity Assay

Six groups were established to evaluate the antiviral efficacy of erythrocyte-delivered MMLNPs (Table 4). After 1 h of circulation in the flow chamber, PAMs were collected and seeded in 96-well plates. Cell viability and PRRSV N gene copy number were measured at 12, 24, 36, and 48 h post-infection by CCK-8 assay and qPCR, respectively.

### 3.6. Statistical Analysis

For all experiments involving primary cells, erythrocytes, and serum were isolated from 6 independent PRRSV-negative Landrace piglets (3 biological replicates per experiment, with 2 piglets pooled per replicate) to control for inter-animal variability. All cell isolations and treatments were performed simultaneously under identical environmental conditions to minimize systematic experimental variation. All experiments were performed in triplicate, with four technical replicates each. The experimental unit was defined as a single well of cultured cells for all in vitro assays. Sample size was determined based on standard practice in the field, and no a priori sample size calculation was performed. Experimental units (cell culture wells) were randomly assigned to treatment groups using a random number table. To minimize potential confounders, the order of treatments and measurements was randomized, and cell culture plates were rotated periodically in the incubator to control for temperature variations. Outcome assessments (qPCR, Western blot, CCK-8 assay) and data analysis were performed in a single-blinded manner, where the analyst was unaware of group assignments. Data were expressed as mean ± standard deviation (SD). Normality of data distribution was assessed using the Shapiro–Wilk test, and homogeneity of variances was verified using Levene’s test. All data met the assumptions of normality and equal variance, and were analyzed by one-way ANOVA followed by Tukey’s post hoc test using GraphPad Prism 8. Statistical significance was set at *p* < 0.05 and *p* < 0.01. No data points were excluded from the analysis, and exclusion criteria for data points were not established a priori.

## 4. Results

### 4.1. Optimization of PRRSV Sensitization and Verification of Erythrocyte Immune Adhesion

**Figure 1:**
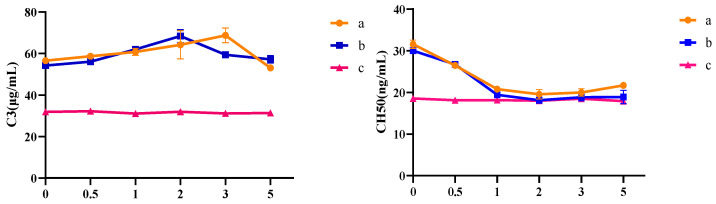
Changes in C3 and CH50 activities in porcine serum incubated with PRRSV at 37 °C. Solid lines represent C3 activity (**left**), and dashed lines represent CH50 activity (**right**). Data are mean ± SD of 3 independent experiments.

ELISA results (Figure 1) showed that PRRSV induced time-dependent complement activation in fresh porcine serum. C3 activity in the experimental group (Group b) peaked at 2 h post-incubation (68.46 ± 5.46 μg/mL), which was significantly higher than that in the blank group (Group a, 64.26 ± 11.84 μg/mL) and inactivated serum control group (Group c, 32.00 ± 1.47 μg/mL, *p* < 0.01). Concurrently, CH50 total complement activity in Group b decreased to the lowest level at 2 h (18.10 ± 0.37 ng/mL), confirming maximum complement consumption. Therefore, 37 °C incubation with fresh porcine serum for 2 h was determined as the optimal PRRSV sensitization condition. Complete C3 and CH50 activity data are provided in Supplementary Tables S1 and S2.

### 4.2. CR1-like-Mediated Immune Adhesion of Porcine Erythrocytes to PRRSV

**Figure 2:**
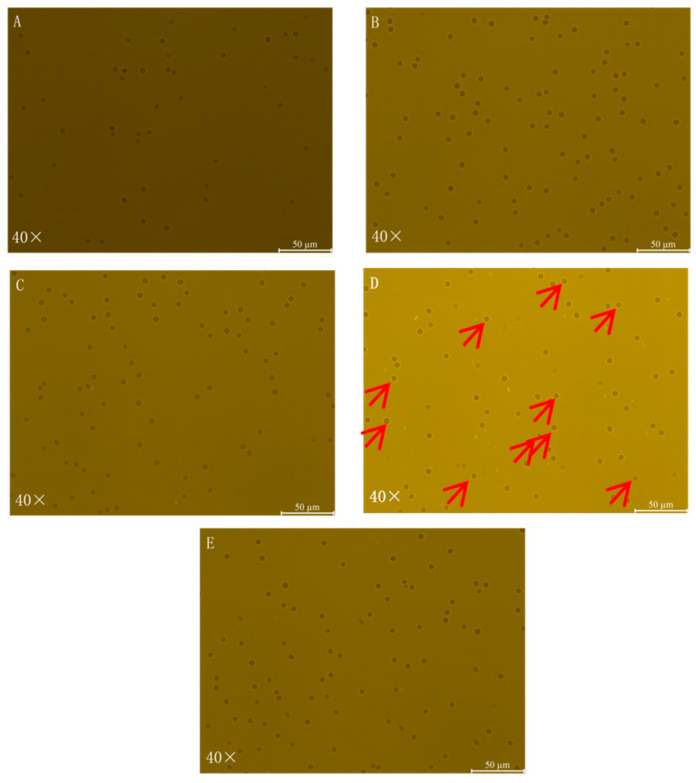
Indirect immunofluorescence observation of erythrocyte adhesion to PRRSV. (**A**) Cell blank group (40× objective magnification). (**B**) Virus control group (40× objective magnification). (**C**) Inactivated serum group (40× objective magnification). (**D**) Sensitized virus group (40× objective magnification). (**E**) Immune blocking group (40× objective magnification). Green fluorescence represents FITC-labeled PRRSV; red arrows indicate PRRSV adhered to porcine erythrocytes via immune adhesion.

**Figure 3:**
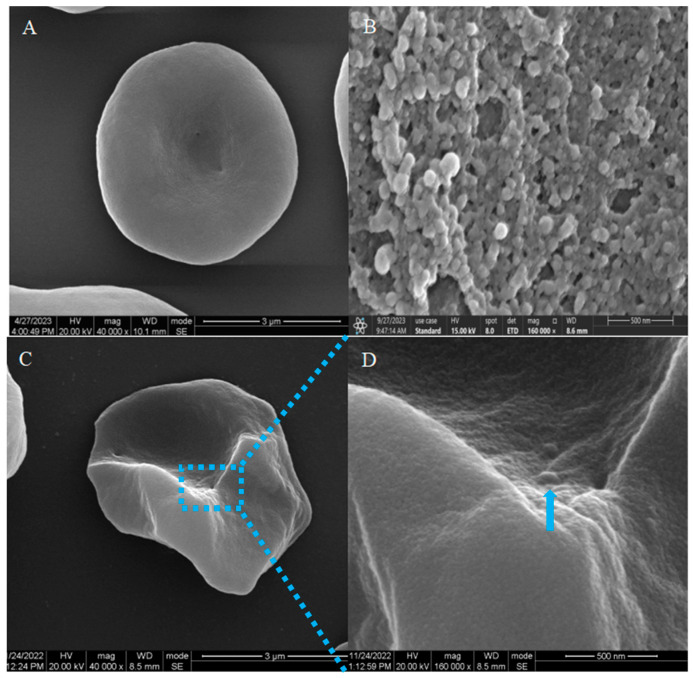
SEM images showing PRRSV immune adherence on porcine erythrocytes. (**A**) Blank erythrocytes; (**B**) Purified PRRSV; (**C**) Erythrocytes bound with serum-opsonized PRRSV; (**D**) Magnification of the framed region in (**C**). (**A**,**C**): 40,000×; (**B**,**D**):160,000×. Blue box denotes enlarged area, blue arrow indicates adhered PRRSV.

**Figure 4:**
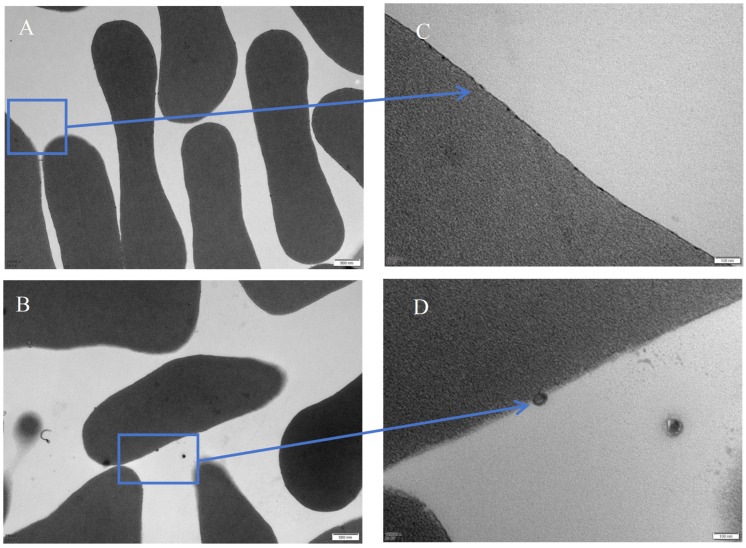
TEM micrographs of PRRSV immune adherence on porcine erythrocytes. Panels (**A**,**B**): blank erythrocytes; (**C**,**D**): erythrocytes bound with serum-opsonized PRRSV; (**A**,**C**) at ×40,000, (**B**,**D**) at ×160,000 magnification. Scale bars: (**A**,**C**) = 500 nm, (**B**,**D**) = 100 nm. Blue boxes mark magnified fields for (**C**,**D**), and the blue arrow denotes surface-adhered PRRSV in (**D**).

Indirect immunofluorescence assay (Figure 2) showed that only the sensitized virus group (Group D) exhibited extensive green fluorescence on erythrocyte surfaces, while no specific fluorescence was observed in the cell blank (Group A), virus control (Group B), inactivated serum (Group C), and immune blocking (Group E) groups. SEM and TEM observations (Figure 3 and Figure 4) further confirmed that spherical PRRSV particles (45–65 nm) tightly adhered to the surface of erythrocytes in Group D, with no non-specific binding detected in other groups.

qPCR quantification revealed that the PRRSV N gene copy number in Group D (1.03 × 10^4^ ± 5.28 × 10^2^) was 82.4-fold higher than that in Group A (1.41 × 10^1^ ± 1.33), 82.4-fold higher than that in Group B (1.25 × 10^2^ ± 1.12 × 10^1^), and 19.3-fold higher than that in Group E (5.33 × 10^2^ ± 2.50 × 10^1^, *p* < 0.01). Western blot results were consistent with qPCR data, showing significantly higher PRRSV N protein expression in Group D than in all other groups (full blots in Supplementary Figure S1). These results collectively demonstrate that CR1-like is a critical receptor mediating the specific adhesion of porcine erythrocytes to fresh serum-sensitized PRRSV.

### 4.3. CR1-like-Mediated Adhesion Promotes PRRSV Infection of PAMs

**Figure 5:**
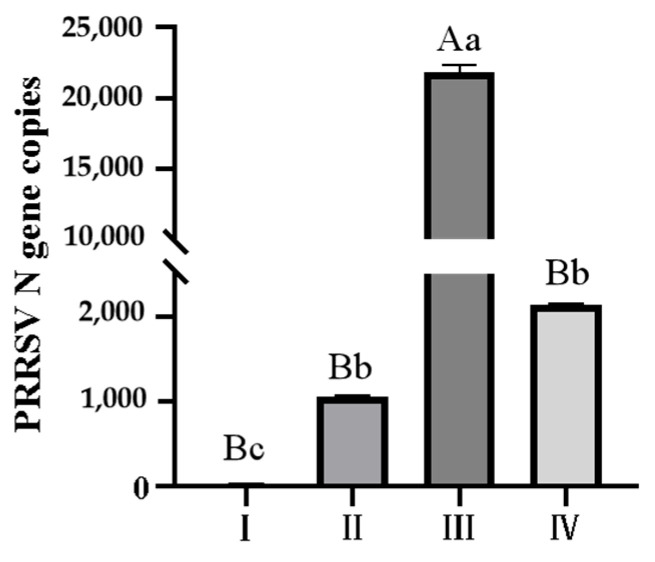
Statistical results of copy number of PRRSV N gene in each group. Capital letters represent the significant difference in the treatment group at the level of 0.01 (*p* < 0.01), while lowercase letters represent the significant difference in the treatment group at the level of 0.05 (*p* < 0.05).

Using the parallel plate flow chamber system that mimics in vivo blood circulation, we investigated the effect of erythrocyte adhesion on PRRSV infection of PAMs. qPCR results (Figure 5) showed that the intracellular PRRSV N gene copy number in the sensitized virus group (Group III, 2.18 × 10^4^ ± 7.68 × 10^2^) was 20.8-fold higher than that in the virus control group (Group II, 1.05 × 10^3^ ± 3.52 × 10^1^, *p* < 0.01).

Notably, blocking CR1-like on PAMs (Group IV) significantly reduced PRRSV infection to a level comparable to Group II (2.13 × 10^3^ ± 4.02 × 10^1^, *p* > 0.05), while the cell blank group (Group I) showed the lowest viral load (3.63 × 10^1^ ± 1.76, *p* < 0.05 vs. Group II and IV). These findings indicate that CR1-like-mediated immune adhesion between erythrocytes and PRRSV significantly enhances viral infection of PAMs. Complete viral copy number data are provided in Supplementary Table S3.

### 4.4. Characterization of MMLNPs and Erythrocyte-Mediated Targeting

#### 4.4.1. Physicochemical Characterization of MMLNPs

**Figure 6:**
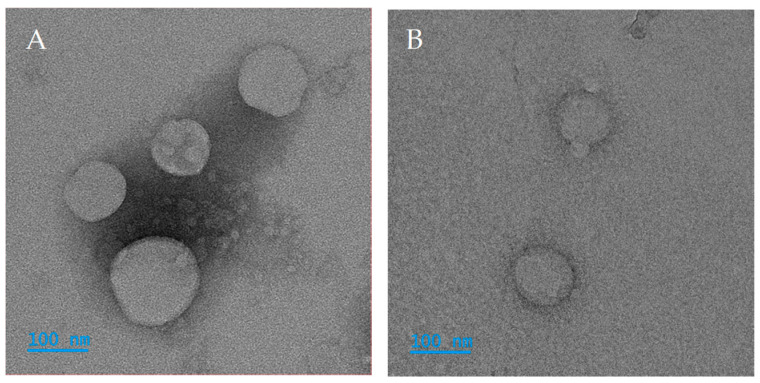
Transmission electron microscope observation of mannose-modified matrine nanoliposomes (MMLNPs) and blank mannose liposomes (MLNPs). (**A**) MMLNPs, scale bar = 100 nm, magnification = 80,000×. (**B**) MLNPs, scale bar = 100 nm, magnification = 80,000×. Both nanoparticles exhibit uniform spherical morphology with smooth surfaces.

The prepared mannose-modified matrine nanoliposomes (MMLNPs) exhibited uniform spherical morphology with smooth surfaces (Table 5, Figure 6). Dynamic light scattering analysis showed an average particle size of 173.01 ± 1.43 nm, polydispersity index (PDI) of 0.221 ± 0.002, and zeta potential of −35.89 ± 0.94 mV. The matrine encapsulation efficiency was 64.62%, with a drug loading rate of 6.22%. Blank mannose liposomes (MLNPs) had a slightly smaller particle size (169.43 ± 5.55 nm) and lower absolute zeta potential (−18.69 ± 2.25 mV).

CCK-8 assay confirmed that both MMLNPs and MLNPs showed no cytotoxicity to PAMs at concentrations up to 2 mg/mL (cell viability > 95%, Supplementary Table S4). Therefore, 1 mg/mL was selected as the working concentration for subsequent experiments.

#### 4.4.2. Erythrocyte Adhesion and PAMs Targeting of MMLNPs

**Figure 7:**
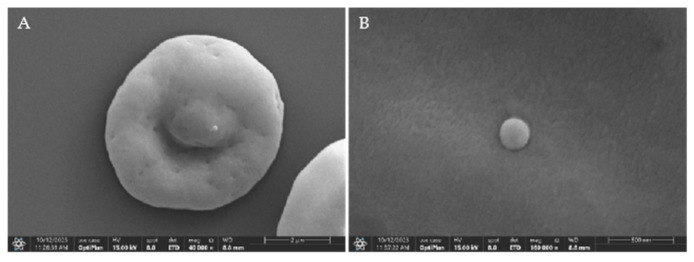
Scanning electron microscope observation of MMLNPs adhered to porcine erythrocyte surface. (**A**) Erythrocytes incubated with inactivated serum-sensitized MMLNPs (no adhesion observed), scale bar = 2 μm, magnification = 15,000×. (**B**) Erythrocytes incubated with fresh serum-sensitized MMLNPs (numerous nanoparticles adhered to the surface), scale bar = 2 μm, magnification = 15,000×.

**Figure 8:**
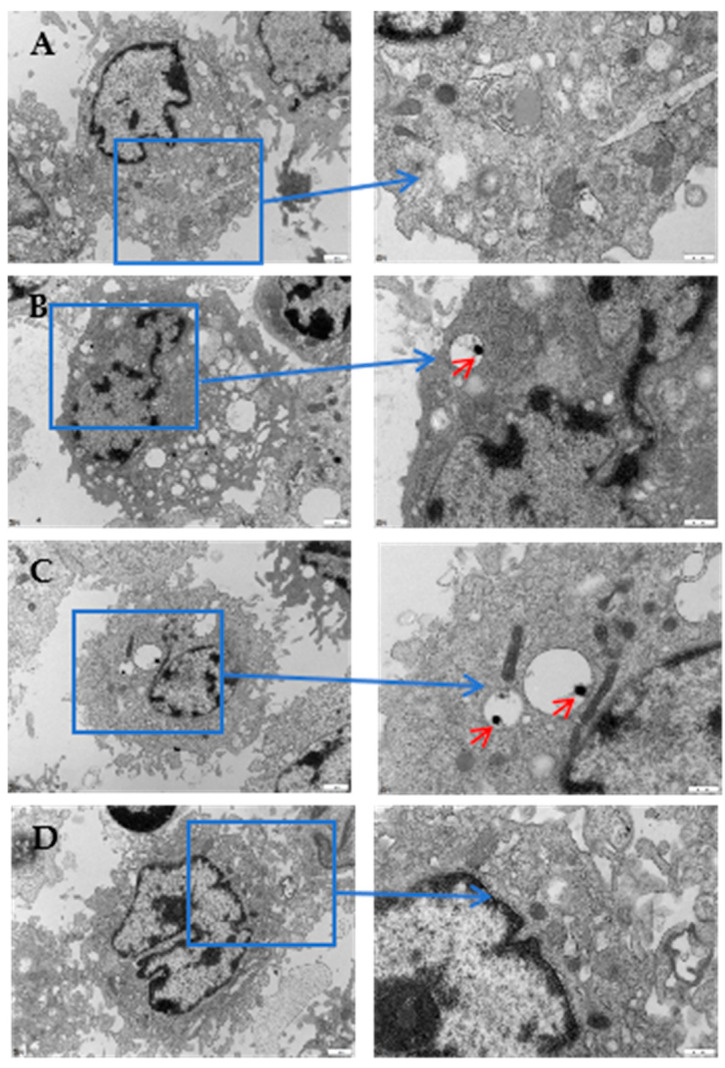
Transmission electron microscope observation of MMLNPs internalized by PAMs. (**A**) Blank control group (no MMLNPs). (**B**) Free MMLNPs group. (**C**) Erythrocyte-delivered MMLNPs group. (**D**) CR1-like blocking + erythrocyte-delivered MMLNPs group. Scale bars: (**A**,**C**) 1 μm; (**B**,**D**) 500 nm. Magnifications: (**A**,**C**) 12,000×; (**B**,**D**) 31,000×. The blue box marks the enlarged area. Red arrows indicate MMLNPs internalized by PAMs.

SEM observation (Figure 7) showed that porcine erythrocytes could effectively adhere to fresh serum-sensitized MMLNPs, while no adhesion was observed with inactivated serum-sensitized MMLNPs. TEM analysis (Figure 8) revealed that erythrocyte-delivered MMLNPs were efficiently internalized by PAMs, with numerous nanoliposomes observed in the cytoplasm. In contrast, almost no MMLNPs were detected in PAMs after CR1-like blocking, verifying the CR1-like-dependent targeting mechanism.

### 4.5. Enhanced Antiviral Activity of Erythrocyte-Delivered MMLNPs

**Figure 9:**
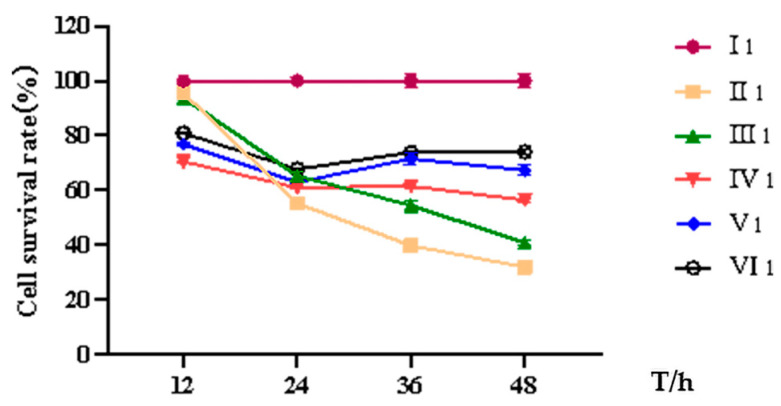
Intracellular antiviral test results.

The in vitro antiviral efficacy of different treatments was evaluated at 12, 24, 36, and 48 h post-infection. CCK-8 results (Figure 9) showed that free matrine exhibited rapid but short-lived antiviral activity, with cell viability decreasing from 93.72% at 12 h to 40.74% at 48 h. In contrast, MMLNPs showed sustained antiviral effects, and erythrocyte-mediated delivery further enhanced this effect.

**Figure 10:**
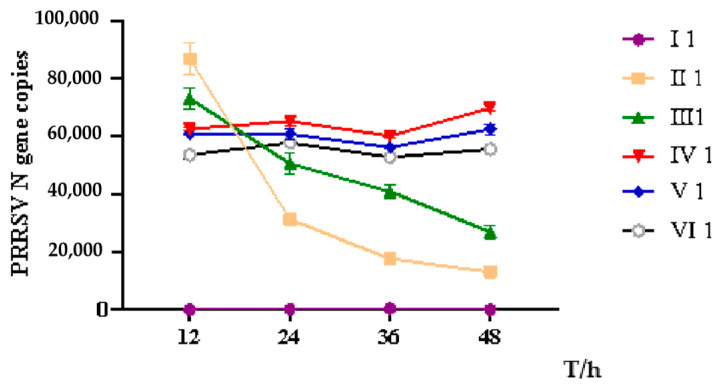
Statistical results of PRRSV N gene copy number at different time points in each group.

At 48 h post-infection, the cell survival rate in the erythrocyte-MMLNPs group (Group VI_1_, 74.01 ± 2.13%) was significantly higher than that in the free matrine group (Group III_1_, 40.74 ± 2.42%, *p* < 0.01) and the MMLNPs alone group (Group V_1_, 67.39 ± 3.66%, *p* < 0.05). qPCR results (Figure 10) were consistent with cell viability data: the PRRSV N gene copy number in Group VI_1_ (5.55 × 10^4^) was 2.05-fold lower than that in Group V_1_ (6.25 × 10^4^, *p* < 0.05) and 4.85-fold lower than that in the virus control group (Group II_1_, 1.31 × 10^4^, *p* < 0.01). Complete antiviral activity data are provided in Supplementary Table S5. These results demonstrate that erythrocyte-mediated delivery significantly improves the intracellular accumulation and sustained antiviral efficacy of MMLNPs against PRRSV.

## 5. Discussion

Complement activation plays a critical role in virus–host interactions, and erythrocyte immune adhesion mediated by complement receptors has been gradually recognized as a key pathway in viral dissemination and pathogenesis [34]. In the present study, we demonstrated that porcine reproductive and respiratory syndrome virus (PRRSV) could activate the porcine serum complement system in a time-dependent manner, and the optimized in vitro sensitization condition was determined as incubation at 37 °C for 2 h. Under this condition, PRRSV was effectively coated with complement fragments, which enabled specific immune adhesion to porcine erythrocytes via CR1-like. Using a dynamic flow chamber system that mimics in vivo hemodynamic shear force, we further confirmed that CR1-like-mediated adhesion significantly enhanced PRRSV infection of porcine alveolar macrophages (PAMs). Moreover, mannose-modified matrine nanoliposomes (MMLNPs) were successfully constructed, and the erythrocyte-mediated targeted delivery system remarkably improved the sustained antiviral efficacy of MMLNPs against PRRSV in vitro. These findings provide novel insights into the mechanism of PRRSV immune evasion and transmission, and offer a promising targeted nano-delivery strategy for PRRSV control.

The complement system exerts three core effector functions against pathogenic microorganisms, including opsonization for phagocytosis, release of inflammatory peptides, and direct lysis via the membrane attack complex (MAC) [34]. Viruses have evolved diverse strategies to interact with or evade the complement system; some viruses harness complement activation to facilitate their own infection and transmission, while others hijack host complement regulatory proteins to resist complement-mediated neutralization [35]. For instance, West Nile Virus, HTLV-1, and HIV can enhance viral infectivity by binding to complement components and interacting with host complement receptors [36]. In this study, ELISA results revealed that PRRSV stimulation triggered a gradual increase in C3 activity and a corresponding decrease in total CH50 complement activity in fresh porcine serum, with the most significant activation observed at 2 h post-incubation. This dynamic change indicated that C3 was cleaved into C3a and C3b, and the complement cascade was fully activated at this time point, which laid a solid foundation for subsequent viral sensitization and erythrocyte adhesion assays.

Erythrocytes are not only responsible for oxygen transport but also participate in immune regulation through various surface receptors, which can bind to exogenous substances and facilitate their clearance [37]. Several pathogens, including malaria parasites, HIV-1, dengue virus, and West Nile Virus, exploit erythrocyte surface receptors to achieve systemic circulation and transmission [38,39,40]. In the present study, immunofluorescence, scanning electron microscopy, and transmission electron microscopy consistently showed that only fresh serum-sensitized PRRSV could tightly adhere to the surface of porcine erythrocytes, while no specific adhesion was detected in the unsensitized, inactivated serum-sensitized, or CR1-like-blocked groups. qPCR and Western blot analysis further verified that the expression levels of PRRSV N gene and protein in the sensitized virus group were extremely significantly higher than those in other groups (*p* < 0.01). These results demonstrated that porcine erythrocytes specifically adhere to complement-sensitized PRRSV in a CR1-like-dependent manner, which is consistent with previous findings in our laboratory [41]. The specific binding between complement fragments on the viral surface and CR1-like on erythrocytes is the molecular basis for this immune adhesion, and blocking CR1-like completely abolishes the adhesion effect, confirming its dominant role in this process. The molecular pathway of porcine CR1-like-mediated immune adhesion has been previously identified and validated by our research group in the Pakistan Veterinary Journal [27].

PRRSV primarily replicates in PAMs and causes severe respiratory and reproductive disorders in pigs, resulting in huge economic losses to the global swine industry. The interaction between PRRSV and host cells is complex and involves multiple receptors and signaling pathways. Macrophages play a central role in erythrocyte homeostasis, including the clearance of aged erythrocytes and the removal of immune complexes bound to erythrocyte CR1 [42]. Notably, the transfer efficiency of immune complexes from erythrocytes to monocytes is significantly enhanced after binding to erythrocyte CR1 [43]. In this study, a parallel plate flow chamber system was used to simulate the in vivo pulmonary capillary microenvironment, and the results showed that the intracellular PRRSV N gene copy number in PAMs was significantly increased after co-culture with erythrocyte-bound sensitized PRRSV. Blocking CR1-like on PAMs markedly reduced viral infection, indicating that CR1-like-mediated immune adhesion promotes PRRSV invasion into PAMs. This dynamic adhesion–infection model more realistically reflects the in vivo viral transmission process compared with static culture systems. This finding extends our previous static culture observations [27] to a more physiologically relevant dynamic system, demonstrating that physiological shear stress does not disrupt CR1-like-mediated adhesion but rather facilitates efficient virus transfer from erythrocytes to PAMs. This suggests that erythrocytes may act as “Trojan horses” for PRRSV dissemination in vivo, which has important implications for understanding PRRSV systemic pathogenesis.

Compared with conventional erythrocyte drug delivery systems based on chemical surface modification, our CR1-like-mediated strategy offers three distinct advantages: (1) Superior targeting specificity—it leverages the natural ligand-receptor interaction between C3b and CR1-like, avoiding non-specific binding to other cells; (2) Enhanced drug loading stability—nanoparticles are bound to the erythrocyte surface via immune adhesion rather than covalent modification, which preserves the structural integrity of both erythrocytes and nanoparticles; (3) Improved biocompatibility—it does not require chemical modification of erythrocyte membranes, thus minimizing damage to erythrocyte physiological functions such as oxygen transport and deformability.

Targeted drug delivery systems exhibit superior advantages over conventional formulations, including site-specific delivery, reduced dosage, and minimized systemic toxicity [44]. Mannose receptors are highly expressed on PAMs and serve as ideal targets for macrophage-specific drug delivery [45,46]. Matrine, a natural alkaloid extracted from Sophora flavescens, has been confirmed to exert effective anti-PRRSV activity in vitro in our previous study [47]. In the present study, MMLNPs were successfully prepared with uniform spherical morphology, suitable particle size (173.01 ± 1.43 nm), favorable zeta potential, and high encapsulation efficiency (64.62%). The particle size and surface charge of MMLNPs met the requirements for efficient cellular uptake and in vivo circulation. Specifically, the average particle size of 173.01 nm falls within the optimal range (100–200 nm) for systemic circulation: particles smaller than 100 nm are rapidly cleared by renal filtration, while those larger than 200 nm are prone to non-specific uptake by the reticuloendothelial system (RES) in the liver and spleen. The highly negative zeta potential (−35.89 mV) confers excellent colloidal stability by preventing nanoparticle aggregation through electrostatic repulsion, and also reduces non-specific protein adsorption (opsonization) in the bloodstream, thereby prolonging circulation half-life. Cytotoxicity assays confirmed that MMLNPs had no obvious toxic effects on PAMs at the tested concentrations. Morphological observations revealed that porcine erythrocytes could adhere to complement-sensitized MMLNPs and efficiently deliver them into PAMs via the CR1-like pathway, and the delivery efficiency was significantly higher than that of free MMLNPs. In vitro antiviral assays showed that erythrocyte-mediated MMLNPs exerted more durable and potent anti-PRRSV efficacy than free matrine and unmodified nanoliposomes, especially at 48 h post-infection (*p* < 0.05). The differential kinetic profiles of free matrine and MMLNPs can be attributed to their distinct cellular uptake and release mechanisms. Free matrine is a small lipophilic molecule that rapidly diffuses across cell membranes, resulting in high intracellular concentrations and rapid antiviral activity within the first 12 h. However, small molecules are also rapidly effluxed from cells via ABC transporters and metabolized by intracellular enzymes, leading to a sharp decline in efficacy over time. In contrast, MMLNPs are internalized by PAMs primarily through clathrin-mediated endocytosis, a slower process that results in delayed onset of action. Once inside the cells, the nanoliposomes gradually release matrine through lipid bilayer degradation, maintaining sustained intracellular drug concentrations for up to 48 h. Furthermore, erythrocyte-mediated delivery enhances the accumulation of MMLNPs in PAMs, further amplifying their sustained antiviral effect. These results are consistent with previous studies showing that mannose modification and targeted delivery systems can enhance cellular uptake and antiviral activity [48,49]. It should be noted that this study did not investigate whether nanoparticle delivery alters the molecular mechanism of matrine against PRRSV. Based on previous studies [6,7,8,9,10], we hypothesize that matrine released from MMLNPs still exerts its antiviral effect by suppressing N protein expression, Nsp9 activation, and NLRP3 inflammasome-mediated inflammation. However, further studies are needed to confirm this and explore whether the nanocarrier itself has any synergistic antiviral activity, which represents a limitation of the current work.

This study has several limitations that need to be addressed in future research. Firstly, all findings were obtained from in vitro cell experiments, and the in vivo performance of the erythrocyte-MMLNPs delivery system requires comprehensive validation in pig models. For translational application, three key aspects need to be addressed: (1) the stability of complement-sensitized MMLNPs in systemic circulation, where host complement regulatory proteins may rapidly degrade surface-bound C3b; (2) the potential for off-target binding to other CR1-like-expressing cells; (3) the biosafety profile of repeated administration, particularly regarding erythrocyte lifespan and systemic complement activation. Notably, erythrocyte-based delivery systems have been safely evaluated in multiple human clinical trials [31], supporting the feasibility of translating this approach to veterinary medicine. However, several potential clinical risks need to be carefully evaluated. Firstly, loading nanoparticles onto erythrocytes may affect their normal physiological functions, including oxygen transport capacity and membrane deformability, which could impair microcirculation. Secondly, complement activation by serum-sensitized nanoparticles may trigger systemic inflammatory responses, although our in vitro cytotoxicity assays showed no obvious adverse effects. Thirdly, only one PRRSV strain (JS-1) was used in this study, and the antiviral effect against different genotypes of PRRSV needs further evaluation. Fourthly, the detailed molecular mechanism of mannose receptors in the antiviral process and the optimization of drug loading capacity of MMLNPs require in-depth investigation. In addition, the long-term effects and potential side effects of targeted nano-drugs in vivo need to be systematically assessed. Beyond PRRSV, this erythrocyte CR1-like-mediated targeted delivery strategy has broad implications for the treatment of other macrophage-tropic swine viruses, including African swine fever virus (ASFV) and swine influenza virus (SIV). Both ASFV and SIV primarily replicate in macrophages and have been shown to interact with the complement system [15], suggesting that they may also exploit erythrocyte immune adhesion for systemic dissemination. Therefore, the platform developed in this study could be readily adapted to deliver antiviral agents against these devastating pathogens by simply changing the encapsulated drug, providing a versatile approach for combating multiple swine viral diseases.

In conclusion, this study clarifies that PRRSV can activate the porcine complement system and be sensitized to adhere to porcine erythrocytes via CR1-like, which in turn promotes viral infection of PAMs under dynamic physiological conditions. Furthermore, the constructed erythrocyte-mediated MMLNPs targeted delivery system significantly enhances the in vitro anti-PRRSV efficacy of matrine. This work not only expands the understanding of PRRSV pathogenesis and immune evasion mechanisms but also provides a novel targeted therapeutic strategy for the prevention and control of PRRSV. However, several critical issues must be addressed before clinical translation, including optimizing the stability of complement-sensitized nanoparticles in systemic circulation, evaluating the long-term biosafety of repeated administration, and scaling up the production of MMLNPs under good manufacturing practice (GMP) conditions. Addressing these challenges will be essential for translating this novel strategy into practical clinical applications for PRRS control.

## 6. Conclusions

**Figure 11:**
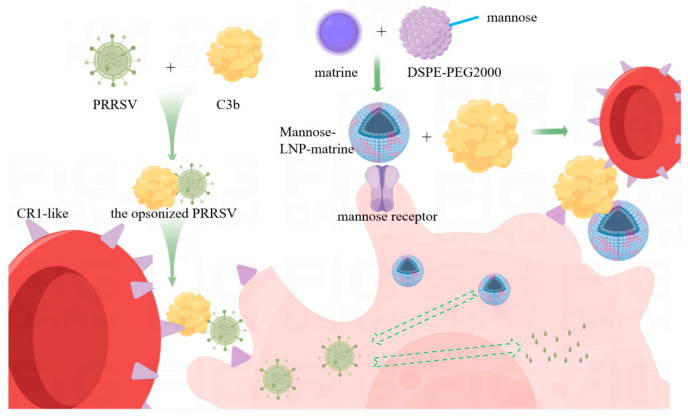
Schematic diagram of CR1-like-mediated immune adhesion of porcine erythrocytes to PRRSV and targeted delivery of anti-PRRSV nanodrugs. CR1-like receptors are expressed on both erythrocytes and PAMs.

This in vitro study confirms that CR1-like-mediated immune adhesion is a key mechanism underlying PRRSV infection of PAMs. Harnessing this natural pathway significantly enhances the targeted delivery and intracellular antiviral efficacy of mannose-modified matrine nanoliposomes. These findings provide a strong preclinical foundation for future in vivo validation in pig models, which will be essential for translating this novel strategy into clinical applications for PRRS control. The corresponding working model is depicted in Figure 11.

## Figures and Tables

**Table 1:** Grouping for PRRSV sensitization condition optimization.

Group ID	Treatment
a (Blank)	Fresh porcine serum + PBS (1:20)
b (Experimental)	Fresh porcine serum + PRRSV (1:20)
c (Control)	Inactivated porcine serum + PRRSV (1:20)

**Table 2:** Grouping for erythrocyte immune adhesion assay.

Group ID	Treatment
A (Cell blank)	Erythrocytes + PBS
B (Virus control)	Erythrocytes + untreated PRRSV
C (Inactivated serum)	Erythrocytes + inactivated serum-sensitized PRRSV
D (Sensitized virus)	Erythrocytes + fresh serum-sensitized PRRSV
E (Immune blocking)	Erythrocytes pre-incubated with anti-CR1-like antibody (100 μL/5 mL cells, 37 °C 2 h) + fresh serum-sensitized PRRSV

**Table 3:** Grouping for PRRSV infection assay in flow chamber.

Group ID	Treatment
I (Cell blank)	PAMs + PBS circulation
II (Virus control)	PAMs + PRRSV circulation
III (Sensitized virus)	PAMs + erythrocyte-bound sensitized PRRSV circulation
IV (Immune blocking)	PAMs pre-incubated with anti-CR1-like antibody (10 μL/slide, 37 °C 2 h) + erythrocyte-bound sensitized PRRSV circulation

**Table 4:** Grouping for in vitro antiviral assay.

Group ID	Treatment
I_1_ (Blank control)	PAMs + PBS circulation
II_1_ (Virus control)	PAMs + PRRSV + erythrocytes circulation
III_1_ (Free matrine)	PAMs + PRRSV + free matrine circulation
IV_1_ (MLNPs)	PAMs + PRRSV + MLNPs + erythrocytes circulation
V_1_ (MMLNPs alone)	PAMs + PRRSV + MMLNPs circulation
VI_1_ (Erythrocyte-MMLNPs)	PAMs + PRRSV + erythrocyte-bound MMLNPs circulation

**Table 5:** MLNPs and MMLNPs particle size and potential detection results.

	Hydrodynamic Size (nm)		PDI		Zeta Potential (mV)	
	MLNPs	MMLNPs	MLNPs	MMLNPs	MLNPs	MMLNPs
Test 1	175.76	170.29	0.168	0.221	−21.14	−35.45
Test 2	167.16	174.29	0.171	0.219	−16.70	−35.39
Test 3	165.37	174.44	0.173	0.224	−18.24	−36.83
mean	169.43 ± 5.55	173.01 ± 1.43	0.171 ± 0.002	0.221 ± 0.002	−18.69 ± 2.25	−35.89 ± 0.94

## Data Availability

The original contributions presented in this study are included in the article and its Supplementary Materials. Further inquiries can be directed to the corresponding author.

## Supplementary Materials

The following supporting information can be downloaded at https://www.mdpi.com/article/10.3390/vetsci13060555/s1: Figure S1: Western Blot images of PRRSV N protein expression; Figure S2. PRRSV N gene qPCR amplification curves; Table S1. Complete C3 activity data at different time points; Table S2. Complete CH50 activity data at different time points; Table S3. PRRSV N gene copy number in erythrocyte adhesion assay; Table S4. MMLNPs and MLNPs physicochemical properties; Table S5. Complete cell viability data of MMLNPs and MLNPs at 72 h; Table S6. Complete antiviral activity data; Table S7. Pairwise comparison P values for antiviral assay at 48 h.
