# Supplementary material for: Porcine Erythrocyte–PRRSV Interactions: Implications for Targeted Nanodrug Delivery
Source: Vet Sci. 2026 Jun 4;13(6):555. doi: 10.3390/vetsci13060555 (PMC13307653; doi:10.3390/vetsci13060555)
Supplement: Supplementary file 1 [file vetsci-13-00555-s001.zip › vetsci-4346646-supplementary.pdf]

Supplementary Materials

Contents

S1 Supplementary Methods
S2 Supplementary Methodological Validation Data
S3 Supplementary Figures
S4 Supplementary Tables
S5 Raw Data Availability Statement

S1 Supplementary Methods

S1.1 Isolation of Porcine Alveolar Macrophages (PAMs)

PAMs were isolated from 30-day-old PRRSV-negative Landrace piglets by bronchoalveolar lavage. Briefly, piglets were euthanized by intravenous injection of pentobarbital sodium (100 mg/kg body weight), and the lungs were aseptically removed. The lungs were lavaged 5 times with 50 mL pre-cooled PBS containing 1% penicillin-streptomycin. The lavage fluid was filtered through 8-layer sterile gauze and centrifuged at 1500 rpm for 5 min at 4°C. The cell pellet was resuspended in RPMI-1640 medium containing 10% fetal bovine serum and seeded in 6-well plates at a density of 1.2×10^7 cells/mL. Non-adherent cells were removed after 2 h incubation at 37°C with 5% CO_2, and adherent PAMs were used for subsequent experiments.

S1.2 Detailed Preparation of MMLNPs and MLNPs

MMLNPs: 200 mg SPC, 30 mg cholesterol, 20 mg DSPE-PEG-mannose (PEG 2000) and 20 mg matrine were dissolved in 5 mL chloroform. The organic solvent was removed by rotary evaporation at 45°C under reduced pressure (0.08 MPa) to form a uniform thin lipid film. The film was hydrated with 5 mL ultrapure water at 40°C for 30 min, followed by sonication at 40 W for 2 min (2 s on/2 s off) in an ice bath. The suspension was extruded 10 times through a 200 nm polycarbonate membrane using a liposome extruder. Unencapsulated matrine was removed by dialysis against ultrapure water for 6 h (molecular weight cutoff: 10 kDa, buffer changed every 2 h). The final MMLNPs solution was lyophilized with 5% sucrose as a cryoprotectant and stored at -20°C.

MLNPs: Prepared identically to MMLNPs without the addition of matrine.

S1.3 Flow Chamber System Parameters

A parallel plate flow chamber system (GlycoTech, USA) was used to simulate in vivo blood circulation. The shear force was calculated using the formula: $\tau = 6\mu Q/(wh^2)$, where μ = dynamic viscosity of medium (0.00075 Pa·s), Q = flow rate (0.4 mL/min), w = channel width (0.01 m), h = channel height (0.00025 m). The calculated shear force was 5.3 dynes/cm², which matches the physiological shear force in porcine pulmonary capillaries.

S1.4 Self-made Antibody Validation

Mouse anti-porcine CR1-like monoclonal antibody (clone 2B11) was prepared in our laboratory (Patent No. ZL201410308534.0). The antibody titer was determined by indirect ELISA as 1:12800. Western Blot analysis showed a single specific band at ~160 kDa in porcine erythrocyte membrane protein, with no cross-reactivity with other porcine serum proteins.

S1.5 Detailed Statistical Analysis Protocol

All experiments were performed in 3 independent biological replicates, with 4 technical replicates per biological replicate. Data were tested for normality using the Shapiro-Wilk test and for homogeneity of variance using Levene's test. One-way ANOVA followed by Tukey's post-hoc test was used for multiple comparisons. Statistical analysis was performed using GraphPad Prism 8.0. Statistical significance was set at $P < 0.05$ and $P < 0.01$.

S2 Supplementary Methodological Validation Data

S2.1 RNA Quality Control Results

Sample	RNA Concentration (ng/μL)	A260/A280	A260/A230
Cell blank (A)	33.5	1.78	0.15
Virus control (B)	252.3	1.78	0.31
Inactivated serum (C)	49.2	1.76	0.05
Sensitized virus (D)	45.6	1.84	0.05
Immune blocking (E)	145.7	1.76	0.18

S2.2 Standard Curves

- Porcine C3: $Y = 0.0218X + 0.0485$, $R^2 = 0.9965$, linear range: 0-80 μg/mL
- Porcine CH50: $Y = 0.1154X + 0.076$, $R^2 = 0.9978$, linear range: 0-20 ng/mL
- Matrine: $Y = 8.704X - 0.003$, $R^2 = 0.9957$, linear range: 0.0125-0.0625 mg/mL
- PRRSV N gene: $Y = -3.339X + 34.688$, $R^2 = 1.000$, amplification efficiency = 100%

S2.3 qPCR Validation

- Melting curve analysis: A single sharp peak at 85.5°C for PRRSV N gene, no non-specific amplification or primer-dimer formation
- Amplification efficiency: 100% ± 2.3% across all runs
- Intra-assay coefficient of variation: < 2%
- Inter-assay coefficient of variation: < 3.5%

S2.4 PRRSV $TCID_{50}$ Determination

Virus Dilution	Positive Wells	Negative Wells	CPE Rate (%)
10^{-1}	6	0	100
10^{-2}	5	1	91.67
10^{-3}	3	3	60
10^{-4}	2	4	27.3
10^{-5}	1	5	7.14
10^{-6} to 10^{-9}	0	6	0

$TCID_{50} = 10^{-3.3}$/mL (calculated by Reed-Muench method)

S3 Supplementary Figures

Figure S1. Western Blot images of PRRSV N protein expression

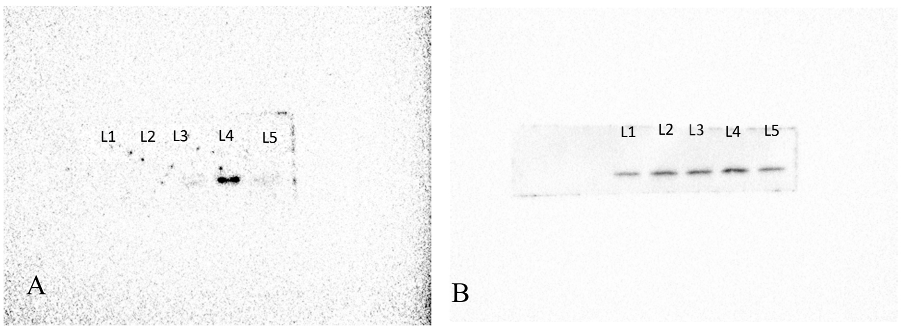

(A): Lane 1: Cell blank ; Lane 2: Virus control Lane 3: Inactivated serum; Lane 4: Sensitized virus; Lane 5: Immune blocking .(B) β-actin (42 kDa) was used as loading control.

Figure S2. PRRSV N gene qPCR amplification curves

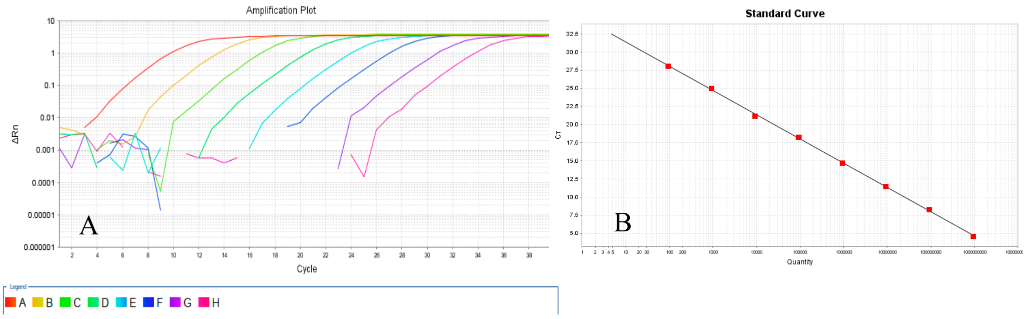

(A) Amplification curve of PRRSV N gene recombinant plasmid; (B) Standard curve of PRRSV N gene recombinant plasmid.

S4 Supplementary Tables

Table S1. Complete C3 activity data at different time points (Mean ± SD, μg/mL)

Time (h)	Blank (a)	Experimental (b)	Control (c)
0	56.54 ± 1.28	54.24 ± 0.83	31.87 ± 1.03
0.5	58.68 ± 0.87	56.08 ± 2.10	32.26 ± 1.87
1	60.82 ± 2.89	62.04 ± 0.92	31.11 ± 0.58
2	64.26 ± 11.84	68.46 ± 5.46\\	32.00 ± 1.47
3	68.77 ± 6.10	59.44 ± 2.45	31.18 ± 0.74
5	53.10 ± 2.55	57.23 ± 3.24	31.37 ± 1.48

$P < 0.01$ vs groups a and c at the same time point

Table S2. Complete CH50 activity data at different time points (Mean ± SD, ng/mL)

Time (h)	Blank (a)	Experimental (b)	Control (c)
0	31.54 ± 1.76	30.02 ± 1.01	18.57 ± 0.88
0.5	26.49 ± 0.81	26.73 ± 1.37	18.11 ± 0.74
1	20.77 ± 1.36	19.43 ± 0.70	18.15 ± 0.51

2	19.55 ± 1.96	18.10 ± 0.37\\	18.02 ± 0.80
3	19.98 ± 1.59	18.86 ± 1.55	18.48 ± 0.83
5	21.67 ± 0.85	18.89 ± 2.81	17.93 ± 0.57

P < 0.01 vs group a at the same time point

Table S3. PRRSV N gene copy number in erythrocyte adhesion assay (Mean ± SD)

Group	Copy Number	Fold Change vs Group A
A (Cell blank)	1.41×10^{1} ± 1.33	1.00
B (Virus control)	1.25×10^{2} ± 1.12×10^{1}	8.87
C (Inactivated serum)	6.50×10^{2} ± 2.71	46.10
D (Sensitized virus)	1.03×10^{4} ± 5.28×10^{2}\\	730.50
E (Immune blocking)	5.33×10^{2} ± 2.50×10^{1}	37.80

P < 0.01 vs all other groups

Table S4. MMLNPs and MLNPs physicochemical properties (3 replicates)

Parameter	MLNPs	MMLNPs
Particle size (nm)	169.43 ± 5.55	173.01 ± 1.43
PDI	0.171 ± 0.002	0.221 ± 0.002
Zeta potential (mV)	-18.69 ± 2.25	-35.89 ± 0.94
Encapsulation efficiency (%)	-	64.62 ± 1.28
Drug loading rate (%)	-	6.22 ± 0.15

Table S5. Complete cell viability data of MMLNPs and MLNPs at 72 h (Mean ± SD, %)

Concentration (mg/mL)	MLNPs	MMLNPs

0	100.00 ± 6.43	100.00 ± 4.83
0.0625	98.65 ± 10.44	104.49 ± 6.78
0.125	97.00 ± 13.42	103.96 ± 5.99
0.25	101.13 ± 10.07	105.77 ± 5.44
0.5	104.56 ± 19.60	106.93 ± 3.65
1	107.87 ± 5.34	108.03 ± 13.17
2	105.89 ± 12.38	100.96 ± 10.47

Table S6. Complete antiviral activity data

(A) Cell viability (Mean ± SD, %)

Group	12 h	24 h	36 h	48 h
I_1 (Blank control)	100.00 ± 3.27	100.00 ± 2.97	100.00 ± 5.29	100.00 ± 4.51
II_1 (Virus control)	95.51 ± 1.20	55.19 ± 0.88	39.72 ± 0.85	31.86 ± 1.37
III_1 (Free matrine)	93.72 ± 0.25	65.21 ± 2.80*	54.50 ± 3.80*	40.74 ± 2.42*
IV_1 (MLNPs)	70.42 ± 0.37	60.99 ± 0.58	61.44 ± 1.96*	56.37 ± 1.14*
V_1 (MMLNPs alone)	76.84 ± 0.95	62.86 ± 0.59	71.46 ± 4.19\\	67.39 ± 3.66\\
VI_1 (Erythrocyte-MMLNPs)	80.89 ± 0.59	67.64 ± 1.50*	73.87 ± 1.08\\	74.01 ± 2.13\\##

P < 0.05, P < 0.01 vs II_1 at the same time point; ##P < 0.05 vs V_1 at 48 h

(B) PRRSV N gene copy number (Mean ± SD)

Group	12 h	24 h	36 h	48 h

I_1 (Blank control)	1.33	1.22×10^{1}	9.89	0.99
II_1 (Virus control)	8.69×10^{4}	3.13×10^{4}	1.76×10^{4}	1.31×10^{4}
III_1 (Free matrine)	7.31×10^{4}*	5.06×10^{4}	4.09×10^{4}*	2.69×10^{4}*
IV_1 (MLNPs)	6.25×10^{4}*	6.52×10^{4}	6.01×10^{4}	6.98×10^{4}
V_1 (MMLNPs alone)	6.08×10^{4}*	6.08×10^{4}	5.62×10^{4}*	6.25×10^{4}*
VI_1 (Erythrocyte-MMLNPs)	5.35×10^{4}\\	5.78×10^{4}*	5.28×10^{4}\\	5.55×10^{4}\\##

P < 0.05, P < 0.01 vs II_1 at the same time point; ##P < 0.05 vs V_1 at 48 h

Table S7. Pairwise comparison P values for antiviral assay at 48 h

Group Comparison	Cell Viability	Viral Copy Number
II_1 vs III_1	0.002	0.021
II_1 vs IV_1	<0.001	0.562
II_1 vs V_1	<0.001	0.018
II_1 vs VI_1	<0.001	0.003
V_1 vs VI_1	0.032	0.027

S5 Raw Data Availability Statement

All raw data generated during this study are available from the corresponding author upon reasonable request, including:

1. Full uncropped Western Blot images
2. Original qPCR amplification and melting curve files
3. Raw absorbance data from all ELISA and CCK-8 assays
4. Original SEM and TEM image files

5. Complete statistical analysis output files
